# New Antidepressant Prescriptions Before, During, and After the COVID-19 Pandemic: Sex and Age Differences in a Population-Based Ecological Study

**DOI:** 10.3390/healthcare13050502

**Published:** 2025-02-26

**Authors:** Monica Martinez-Cengotitabengoa, Monike Sanchez-Martinez, Andoni Sanchez-Martinez, Daniel Long-Martinez, Daisy Dunford, Paula Revuelta, Enrique Echevarria, Begoña Calvo

**Affiliations:** 1 Faculty of Pharmacy, University of the Basque Country UPV/EHU, 01006 Vitoria-Gasteiz, Spainb.calvo@ehu.eus (B.C.); 2 Osakidetza Basque Health Service, 48902 Barakaldo, Spain; paularevuelta@gmail.com; 3 Psychology School, Universidad Publica de Navarra, 31009 Pamplona, Spain; monikessanchez@gmail.com; 4 Psychology School, Universidad Pontificia de Salamanca, 37002 Salamanca, Spain; andonisanmar22@gmail.com; 5 Psychology Clinic of East Anglia, Norwich NR1 4AA, UK; danimartinezcengotitabengoa@gmail.com (D.L.-M.); ddunford@gmail.com (D.D.)

**Keywords:** COVID-19, pandemics, antidepressive agents, drug prescriptions, sex differences, age groups

## Abstract

**Purpose:** It is well established that the COVID-19 pandemic has increased the incidence of depressive symptoms in the general population. So far, there are no studies analyzing how this pandemic has affected the prescription of antidepressants. **Methods:** We retrospectively estimated the incidence of new cases of depression by analyzing new prescriptions of antidepressive agents over a period of 6 years distributed in three periods of 2 years each: pre-pandemic, pandemic, and post-pandemic, running from March 2018 to February 2024. This research was conducted in a healthcare area with 130,000 individuals in Spain. **Results:** New prescriptions for antidepressants increased significantly during the pandemic and even more in the post-pandemic period, with the increase being greater in women than in men. During the pandemic period, the increased prescription for antidepressants was specifically due to an increase among people under 20 years of age, while in the post-pandemic period, the increase has occurred in all age groups. In the subgroup analysis, we found that the pandemic period exclusively affected the number of women aged 40 or under who were prescribed a new antidepressant, while in the post-pandemic period, there has been a significant increase in the number of both male and female patients with a new antidepressant in all age groups compared to the two previous periods, except in young men aged up to 20 years. **Conclusions:** Our study highlights the importance of addressing the mental health of the population in parallel with addressing physical problems and adapting health systems accordingly.

## 1. Introduction

Since December 2019, the COVID-19 epidemic has become a pandemic with unprecedented consequences in contemporary history, and authorities have had to take exceptional measures to contain its rapid expansion, such as home confinement, closure of non-essential establishments, instituted surveillance [1].

Since then, both the high morbidity and mortality of the disease and the measures restricting the movement of people have drastically influenced the incidence of mental health problems in the population [2,3,4]. The effects of the COVID-19 pandemic on mental health issues, such as depression, are widely documented [5,6,7]. A WHO report points out that the COVID-19 pandemic has increased the prevalence of depression by between 25% and 27% [2].

Furthermore, the difficulty accessing the health system, which had devoted most of its resources to tackling the pandemic, made it difficult to access non-pharmacological treatments for mental illness, thereby favoring the prescription of psychotropic drugs [8,9]. Since the first months of the pandemic, a statistically significant increase of approximately 20% has been noted in new antidepressant (AD) prescriptions compared to the period immediately preceding the pandemic [10].

Many studies have explored the increase in depressive symptoms during the most restrictive period of the pandemic [3,4,11]; however, there is still little research on the residual impact of the pandemic on the population’s mood, and some experts recommend that further research should be conducted on the long-term effects of the pandemic on AD prescription [12].

In our environment, the most frequent treatment for depression is pharmacological. In the Basque Country, the prescription for ADs increases significantly with age and in women, reaching 21% of women and 9% of men over 65 years of age. ADs are often prescribed due to a lack of other alternatives, insufficient accessibility to psychological therapies, and lack of time. Sometimes, the prescriptions are given in situations of grief, sadness, suffering, and chronic stress where providing psychotherapeutic strategies (such as training on problem solving) or self-care skills could be more appropriate [13].

The objective of this study is to estimate the incidence of depressive symptoms in the general population before, during, and after the COVID-19 pandemic from March 2018 to February 2024.

## 2. Materials and Methods

### 2.1. Setting

We conducted a retrospective population-based study in a healthcare setting in Barakaldo-Sestao, which serves an approximate population of 130,000 and is located in the Basque Country in the north of Spain. The public health system in Spain is a tax-financed universal system that covers all or part of the cost of the medication prescribed to patients depending on their income (the patient pays a percentage ranging between 0 and 60% of the cost). ADs are only dispensed in a community pharmacy when a medical prescription is presented.

### 2.2. Data Collection

The incidence of new cases of depression was estimated based on analysis of new prescriptions for ADs in the whole population in Barakaldo-Sestao over a period of 6 years, distributed in 3 periods of 2 years each: pre-pandemic period (PRE) from 1 March 2018 to 28 February 2020, pandemic period (PAN) from 1 March 2020 to 28 February 2022, and post-pandemic period (POST) from 1 March 2022 to 29 February 2024.

These dates were chosen because March 2022 was when the rapid spread of the infection in Spain began. Only new prescriptions were selected to assess whether the pandemic period had influenced the initiation of new treatments. New prescriptions (presented in %) for AD medications are defined as the percentage of persons in the study population who received a new prescription for an AD for the first time (did not receive it in the previous year). A change in the drug dosage was not considered a new prescription.

Population data were extracted from the electronic prescription system of Osakidetza-Basque Health System, which collects all the pharmacological prescriptions written in the primary care units, extra-hospital mental health centers, and hospital outpatients, all of which are public facilities. Each patient was counted only once, regardless of the number of new prescriptions received throughout the study period.

### 2.3. Statistical Analysis

The Anatomical Therapeutic Chemical (ATC) classification system divides active substances into different groups according to the organ or system on which they act and their therapeutic, pharmacological, and chemical properties. The ATC system is controlled by the WHO Collaborating Centre for Drug Statistics Methodology [14]. The information on all new prescriptions of “antidepressant drugs” according to the ATC classification third level (N06A) was downloaded and presented both in raw form and as a percentage of the reference population. An initial univariate analysis was conducted as it is a valid analysis for studying the behavior of individual variables and evaluating the initial relationship between study variables. It provides statistical validity since the value of the estimates approximates the population. To analyze the variability in the prescription of ADs based on the chosen time period, the odds ratio (OR) with a 95% CI and the Z statistic were calculated, setting the significance level at 5% (*p* < 0.05). Bonferroni correction was conducted to correct for bias of multiple testing in order to ensure the validity of the results (*p* = 0.05/n, where n is the number of comparisons). In order to detect variability in the influence of the pandemic on different subgroups, analyses were carried out on the samples based on sex, age groups, and both factors (sex and age). The SPSS statistical software version 29.0 was used for the analyses.

### 2.4. Ethics Statement

The study complied with European data protection regulations [15], and all data were anonymized before analysis.

## 3. Results

Table 1 shows the distribution of the study population by age and sex during the three time periods analyzed.

**Figure 2:**
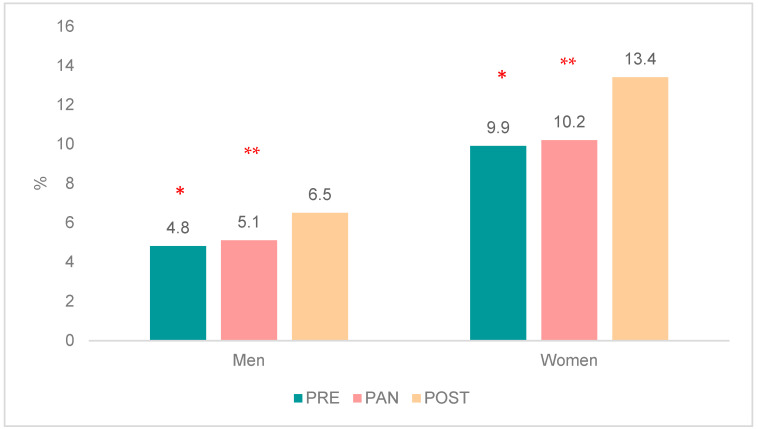
Population (%) with a new prescription for antidepressants according to the patient’s sex. Statistically significant difference between * PANDEMIC and PRE-PANDEMIC periods; ** POST-PANDEMIC AND PANDEMIC periods (* and ** indicate *p*-values < 0.05).

Analyzing the data by sex showed that the increase in new AD prescriptions was statistically significant in men and women both from PRE to PAN (OR ♂ = 1.06, *p* = 0.030; OR ♀ = 1.04, *p* = 0.020) and from PAN to POST (OR ♂ = 1.28, *p* < 0.001; OR ♀ = 1.18, *p* < 0.001) (Figure 2).

In all the periods studied, the percentage of women who received a new prescription for an AD was much higher than the percentage of men.

A comparison of data across different time periods by age group revealed that the increase in new AD prescriptions during the PAN period was primarily driven by prescriptions among individuals under 20 years of age. In contrast, in the POST period, the number of prescriptions increased across all age groups, except those up to 20 years old (Table 2).

Looking at new prescriptions by age and sex subgroups, we found that the PAN period exclusively affected the number of women aged 40 or under who were prescribed a new AD (Table 3).

In the POST period, there has been a significant increase in the number of male and female patients with a new AD in all age groups compared to the two previous periods, except in those up to 20 years old.

The group aged up to 20 years did not see any changes in prescriptions for ADs over the three periods considered.

## 4. Discussion

In the present study, we observed that the 2 years of the COVID-19 pandemic significantly affected first-time antidepressant prescriptions in the public health system (with a potential cause being an increase in depressive symptoms), mainly in women under 40 years of age. In line with these results, a study carried out in Canada reported that women reported worse self-perceived mental health than men during the pandemic period from September to December 2020 [16]. Additionally, some authors have found an increase in AD consumption by young women since the beginning of the pandemic [17,18]. The greater impact on the emotional well-being of young women during the pandemic could be due to the role of caregivers, which is more prevalent in the female population, and the feminization of the care professions in the health sector, which had a high emotional burden during this period [19]. Similar results were reported in the study by Fond et al., who found a higher increase in prescriptions for ADs in the pandemic versus pre-pandemic period in women compared with men in France [20].

Several studies have identified an increase in AD sales during the pandemic [21,22], but we consider that data on new medical prescriptions better reflect the real needs of the patients compared with sales data since these may reflect the oversupply of medicines and other goods detected at the beginning of the pandemic due to fears of shortages. By including the 2 years prior to the pandemic in the study, we can definitively show that the observed increase in the prescription for antidepressants is not due to a continuation of a trend that started prior to the emergence of COVID-19.

Surprisingly, once the COVID-19 pandemic was largely resolved, we observed that men and women of all age groups (except men < 20 years) had a higher incidence of new depressive symptoms, estimated from new AD prescriptions, even though in-person access to health systems was guaranteed or at least more normalized in this period than during the pandemic. We detected a greater long-term impact of the pandemic on the emotional health of the population, which may be due to several factors. Although movement restrictions have eased, the prolonged social isolation suffered by the majority of the population may have had long-lasting consequences on the way people interact and feel connected with others [23]. In addition, the economic impact of the pandemic, such as job loss or financial insecurity, continues to affect the mental well-being of many people [24,25].

Maintaining a sustained state of stress during the pandemic could be one of the main causes of this higher incidence of depressive symptoms in the general population in the long term [26]. In many cases, grief over the loss of loved ones or significant life changes during the pandemic make it impossible to return to previous normality and continue to affect mental health [27].

In our opinion, and as other authors have indicated, it is possible that the pandemic delayed access to health services for those who needed it, and we are seeing the delayed impact on people’s mental health [28]. Research on the association between new AD utilization and the incidence of major depression would improve our understanding of how well AD prescription rates reflect the needs of the population.

In the face of a physical pathology of the notoriety that the COVID-19 pandemic has had worldwide, it is of vital importance to implement a preventive approach to depressive pathology in the population and to monitor the mental health of the population in parallel [29,30]. It is, therefore, important to identify opportunities for public mental health interventions in order to adapt the mental health system to the needs and demands of the population [31], including public policies and mental health strategies.

Based on the experience gained from the pandemic, it is necessary to facilitate the general population’s access to both pharmacological and non-pharmacological therapies in order to prevent an increase in the incidence of depression in the event a similar situation occurs in the future. Likewise, in these situations, more attention should be paid to certain age groups, especially women under 40 years of age, since it has been observed that this group is especially sensitive.

### Study Strengths and Limitations

This study represents a novel approach to the analysis of the long-term effect of the COVID-19 pandemic on the population’s mental health where we comprehensively collected data on all new prescriptions for ADs in a large sample population over a period of 6 years, including the periods before, during, and after the pandemic, highlighting the relevance of real-world data in generating real-world evidence for drug prescription, helping to make health-driven decisions in response to changing public health conditions. Our work highlights specific population groups to which special attention should be paid; however, it has some limitations.

The ecological fallacy is inherent to the type of study carried out, and the existence of uncontrolled confounding variables may mean that population results cannot be extrapolated to the individual level. In addition, univariate analysis limits our understanding of interactions between variables. In order to avoid this fallacy as much as possible, we grouped the samples by age and sex to determine the influence of the pandemic on new AD prescriptions in these subgroups.

Additionally, the collected data only represent prescriptions for ADs in the public system, excluding analysis of all treatments initiated in the private sector.

Furthermore, it should be noted that some antidepressants, in addition to treating depression, are sometimes prescribed to a greater extent (up to 45%) to control other pathologies, such as anxiety symptoms, insomnia, or chronic pain [32]. Due to this limitation, the results of this study should be considered as an estimate of the incidence of anxiety-depressive symptoms in the general population.

Finally, the number of new antidepressant consumers relies on prescriptions; we did not verify whether the patients had collected the ADs from the pharmacy or if they had actually taken them.

## Figures and Tables

**Figure 1:**
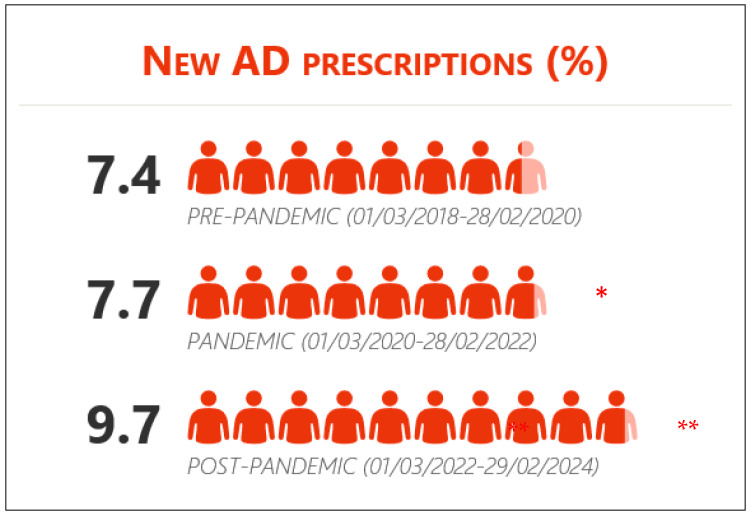
New prescriptions for antidepressants before, during, and after the COVID-19 pandemic (% of population). Statistically significant differences between * PANDEMIC and PRE-PANDEMIC periods; ** POST-PANDEMIC AND PANDEMIC periods.

**Table 1:** Distribution of the population by sex and age in the periods compared: pre-pandemic (PRE), pandemic (PAN), and post-pandemic (POST).

	PRE (*n* = 131,300)		PAN (*n* = 129,260)		POST (*n* = 130,126)	
**Age group (years)**	**N**	**%**	N	%	N	%
0–20	23,495	17.89	23,016	17.80	23,070	17.73
21–40	28,527	21.73	26,832	20.76	26,744	20.55
41–60	42,510	32.38	42,239	32.68	42,540	32.69
61–80	27,761	21.14	28,316	21.91	28,981	22.27
>80	9007	6.86	8857	6.85	8791	6.76
**Sex**						
Female	67,677	51.54	66,586	51.51	67,007	51.49
Male	63,623	48.46	62,674	48.49	63,119	48.51

In the whole sample, new AD prescriptions increased significantly during the pandemic period (PAN) (7.7 vs. 7.4, OR = 1.04, *p* = 0.002) and even more in the post-pandemic period (POST) compared to PAN (9.7 vs. 7.7, OR = 1.28, *p* < 0.001) (Figure 1).

**Table 2:** New antidepressant prescriptions by age group according to the study period.

	PRE 1 March 2018–28 February 2020		PAN 1 March 2020–28 February 2022		POST 1 March 2022–29 February 2024		PAN vs. PRE			POST vs. PAN		
Age (Years)	N	%	N	%	N	%	OR (95% CI)	Z	*p*	OR (95% CI)	Z	*p*
0–20	259	1.1	321	1.4	374	1.6	1.27 (1.08–1.50)	2.834	0.005	1.16 (1.00–1.35)	1.993	0.046
21–40	1653	5.8	1695	6.3	2130	8.0	1.10 (1.02–1.18)	2.577	0.010	1.28 (1.20–1.37)	7.389	<0.001
41–60	3594	8.5	3610	8.5	4728	11.1	1.01 (0.96–1.06)	0.481	0.630	1.34 (1.28–1.40)	12.520	<0.001
61–80	2678	9.6	2741	9.7	3402	11.7	1.00 (0.95–1.06)	0.132	0.894	1.24 (1.18–1.31)	7.952	<0.001
>80	1543	17.1	1616	18.2	1943	22.1	1.08 (1.00–1.17)	1.952	0.051	1.27 (1.18–1.37)	6.376	<0.001

In red are statistically significant differences with a *p*-value < 0.01 after Bonferroni correction.

**Table 3:** Distribution of new antidepressant prescriptions based on sex and age group (% of population).

					PAN vs. PRE			POST vs. PAN		
Age (Years)	Sex	PRE (%)	PAN (%)	POST (%)	OR (95% CI)	Z	*p*	OR (95% CI)	Z	*p*
0–20	♂	0.7	0.8	0.8	1.15 (0.86–1.56)	0.945	0.344	1.08 (0.82–1.44)	0.565	0.572
	♀	1.6	2.1	2.5	1.33 (1.09–1.62)	2.797	0.005	1.20 (1.00–1.43)	1.996	0.045
21–40	♂	4.1	4.4	5.5	1.07 (0.95–1.20)	1.160	0.246	1.28 (1.14–1.43)	4.343	<0.001
	♀	7.5	8.3	10.4	1.11 (1.02–1.21)	2.341	0.019	1.29 (1.19–1.40)	6.059	0.001
41–60	♂	5.7	5.7	7.8	1.01 (0.93–1.10)	0.229	0.819	1.39 (1.28–1.50)	8.354	< 0.001
	♀	11.3	11.4	14.5	1.01 (0.95–1.08)	0.458	0.647	1.32 (1.24–1.39)	9.426	< 0.001
61–80	♂	6.0	6.3	7.8	1.05 (0.94–1.16)	0.871	0.384	1.27 (1.16–1.40)	5.040	< 0.001
	♀	12.7	12.6	15.1	0.99 (0.92–1.06)	0.382	0.702	1.23 (1.15–1.31)	6.315	< 0.001
>80	♂	13.4	15.1	18.3	1.15 (1.00–1.33)	1.942	0.054	1.26 (1.10–1.44)	3.333	< 0.001
	♀	19.1	19.9	24.1	1.05 (0.96–1.15)	1.077	0.282	1.28 (1.17–1.39)	5.417	< 0.001

In red are statistically significant differences after Bonferroni correction.

## Data Availability

The data presented in this study are available on request from the corresponding author.
